# Pharmacokinetics of HDM7006, a high-potency balanced GLP-1/GIP dual agonist, in healthy domestic cats

**DOI:** 10.3389/fvets.2026.1988580

**Published:** 2026-10-28

**Authors:** Zhiping Zhang, Shujun Cui, Chunhua Jiang, Li Teng, Hao Pan, Zhao Dong, Lin Peng, Baojun Wang, Dongzhou Liu

**Affiliations:** Global R&D Center, Huadong Medicine, Zhejiang, Hangzhou, China

**Keywords:** cats, glucagon-like peptide-1 receptor, glucose-dependent insulin-stimulating polypeptide receptor, HDM7006, pharmacokinetics

## Abstract

**Background:**

HDM7006 is a GLP-1R/GIPR dual agonist developed for body weight management in obese or overweight cats.

**Objective:**

To Investigate the pharmacokinetic (PK) characteristics of HDM7006 in cats.

**Animals:**

Forty-eight healthy adult cats were enrolled.

**Methods:**

In the single-dose PK study, 40 cats were divided into five groups receiving 0.1, 0.5, 1.0 or 2.0 mg/kg (SC) or 1.0 mg/kg (intravenous, IV) of HDM7006, respectively. In the multiple-dose PK study, 8 cats were received 0.5 mg/kg of HDM7006 once weekly for five consecutive doses. Plasma samples were collected before and after drug administration to determine PK parameters and evaluate immunogenicity.

**Results:**

No gender differences were observed in system exposure of HDM7006 after IV or SC injection. HDM7006 exhibited a low clearance, a low apparent volume of distribution, and a half-life of 43.7 h in cats after IV injection. Following single SC injection, the median T_max_ ranged from 18.0 to 24.0 h, the T_1/2_ ranged from 39.8 to 47.1 h, and the bioavailability ranged from 42.23 to 78.38%. Linear PK characteristics were observed within the dose range of 0.1–2.0 mg/kg. Anti-drug antibodies (ADAs) were detected only in one male animal at 336 h post-dose in the 2.0 mg/kg dose group. After five consecutive doses of 0.5 mg/kg, steady state was clearly achieved, and no significant accumulation observed; all ADA test results were negative.

**Conclusions and clinical importance:**

HDM7006 exhibits predictable, dose-proportional PK and low immunogenicity, supporting a QW SC regimen in cats, and may offer a long-acting therapeutic option for feline weight management and metabolic regulation.

## 1 Introduction

The GLP-1 receptor is a G protein-coupled receptor widely distributed in multiple organs, such as the gastrointestinal tract, pancreas, heart, and kidneys, with its core function being the regulation of metabolic homeostasis (1–3). Upon activation, the GLP-1 receptor exerts multiple effects through the cAMP signaling pathway: it promotes glucose-mediated insulin release, thereby aiding blood glucose regulation; it delivers satiety signals, delays gastric emptying, and reduces food intake; it exhibits protective effects on organs such as the cardiovascular system and kidneys (4–8).

Glucose-dependent insulinotropic polypeptide (GIP) is an intestinal hormone released following glucose and fat intake. Beyond its well-established effects on the entero-insular axis, activation of the GIP receptor (GIPR) confers multiple metabolic benefits, including improved lipid metabolism and storage, appetite suppression, and weight reduction through its actions in the brain-particularly when combined with long-acting GLP-1 receptor agonists (LAGLP-1RAs) (9–13).

Studies have shown that GLP-1R/GIPR dual agonists exhibit complementary effects (14, 15), achieving synergistic weight loss by activating specific neural circuits in the central nervous system (e.g., the medullary dorsal vagal complex-central amygdala neuropeptide A pathway), while simultaneously promoting insulin secretion, delaying gastric emptying, and suppressing appetite (16, 17). Some dual agonists delay receptor internalization by preferentially targeting signal transduction pathways [e.g., selective activation of the cAMP pathway rather than β-arrestin recruitment (6)], thereby enhancing glucose-lowering and weight-loss effects while reducing adverse effects. Overall, GLP-1R/GIPR dual agonists demonstrate significant advantages in glucose reduction, weight loss, and metabolic improvement (18–21), and their application have expanded to therapeutic areas such as metabolic dysfunction-associated steatohepatitis (MASH) (22), heart failure (23, 24), cognitive dysfunction (including Alzheimer’s disease (AD), Parkinson’s disease (PD), and vascular dementia) (25–27) and osteoarthritis (28–30). Currently, the FDA has approved semaglutide for the treatment of MASH (31), and clinical development of ecnoglutide for obstructive sleep apnea [OSA, NCT07434050 (32) and NCT07387094 (33)] is underway.

The feline GLP-1R and GIPR exhibit high homology with their human counterparts. Studies have demonstrated that feline GLP-1R mRNA is expressed in various peripheral tissues, including the intestine, pancreas, and liver, whereas feline GIPR mRNA is expressed in the stomach and adipose tissue (34). GLP-1 analogs reduce body weight and fasting blood glucose levels in healthy cats, elevate circulating insulin levels, and decrease glucagon concentrations, thereby exerting a hypoglycemic effect (34, 35). Previous studies have also demonstrated that liraglutide can reduce body weight and inhibit food intake in obese cats (36).

Obesity has been officially classified as a disease by the World Small Animal Veterinary Association (WSAVA), contributing to numerous chronic conditions in pets, such as diabetes, cardiovascular diseases, and arthritis (37). Companion animal obesity has reached alarming levels in the United States, presenting a critical veterinary health concern (38, 39). According to a 2025 survey by the Association for Pet Obesity Prevention, 37% of dog owners and 43% of cat owners described their pet as above ideal weight, compared with 35 and 33% in 2024. Despite active weight loss efforts-reported by 56% of dog owners and 53% of cat owners-success rates remained low, with only 28% of dogs and 19% of cats reaching a healthy target weight (40). The rising trend in U. S. pet obesity are part of a global epidemic, studies in Brazil, China, Russia, the United Kingdom, and Spain reporting comparably elevated rates (41, 42). These statistics collectively underscore the need for both preventive and therapeutic approaches to pet obesity. Data from the 2022 China Pet Industry White Paper show that the obesity rate among Chinese pet cats reached 28.1%, while that among pet dogs was 25.2% (43). A 2023 survey by Nestlé Purina reported an overall obesity rate of 51.4% among Chinese pets, with 44.8% for dogs and 58.0% for cats. According to a report by the New Think Tank Industry Research Center, the prevalence of diabetes among pet cats in China is approximately 0.4–0.5% (44). Based on these data, the estimated number of diabetic cats in China is approximately 300,000, corresponding to a market size of approximately 760 million RMB.

As a human therapeutic, HDM7006 (code name: HDM1005) has entered Phase III clinical trials for type 2 diabetes [NCT07394114 (45) and NCT07417306 (46)] and obesity/overweight [NCT07279194 (47)]. Given that HDM7006 exhibited agonistic activity toward feline GLP-1R/GIPR *in vitro* and significant weight-reducing effects *in vivo* pharmacodynamic studies, so now HDM7006 is developing for body weight management in obese or overweight cats. *In vitro* cell agonist activity assays demonstrated that the EC_50_ values of HDM7006 for GLP-1R in humans and cats were 2.67 pM and 7.95 pM, respectively, which were significantly lower than those of Tirezpatide (EC_50_ for human: 12.04 pM; EC_50_ for cat: 379.10 pM). The EC_50_ values of HDM7006 for GIPR in humans and cats were 0.49 pM and 1.74 pM, respectively, which were comparable to those of Tirezpatide (EC_50_ for human: 0.63 pM; EC_50_ for cat: 5.08 pM). In the pharmacodynamic studies for weight reduction in cats, obese cats enrolled in the study were subcutaneously administered low-dose (0.019 mg/kg QW*2, 0.25 mg/kg QW*1, 0.5 mg/kg QW*2), medium-dose (0.075 mg/kg QW*2, 0.50 mg/kg QW*1, 1.0 mg/kg QW*2), or high-dose (0.3 mg/kg QW*2, 1.0 mg/kg QW*1, 2.0 mg/kg QW*2) doses of HDM7006 injection. Compared with the vehicle control group, these treatment groups exhibited dose-dependent reductions in food intake, improvements in body condition score (BCS), and decreases in body weight. At the study endpoint, the weight reduction rates in the low-, medium-, and high-dose groups were 8.42%, 13.71%, and 19.45%, respectively. To support the Phase II and III trials, this present study was conducted to investigate the bioavailability of HDM7006 following subcutaneous or intravenous administration in cats, as well as its pharmacokinetic characteristics following single-dose (0.1–2.0 mg/kg) and repeated-dose (once weekly, 0.5 mg/kg) subcutaneous administrations.

## 2 Materials and methods

### 2.1 Study design

The single-dose intravenous group (G1) received a dose of 1.0 mg/kg with a delivery volume of 0.25 mL/kg. The four single-dose subcutaneous groups (G2-G5) received doses of 0.1 (G2), 0.5 (G3), 1.0 (G4), and 2.0 (G5) mg/kg, with corresponding delivery volumes of 0.025, 0.125, 0.25, and 0.5 mL/kg, respectively. The repeated-dose subcutaneous administration group (G6) received 0.5 mg/kg with a delivery volume of 0.125 mL/kg, administered once-weekly for a total of 5 doses. The intravenous group received the drug via posterior limb veins, while the subcutaneous groups received the drug via the cervical and dorsal regions.

The PK blood sample collection time points for each dose group were as follows:

Single-dose intravenous group (G1): Before administration (within 60 min prior to administration) and at 0.0833, 0.5, 2, 4, 8, 12, 24, 36, 48, 72, 96, 120, 144, 192, and 240 h post-administration.

Single-dose subcutaneous groups (G2-G5): Before administration (within 60 min prior to administration) and at 0.5, 2, 4, 8, 12, 24, 36, 48, 72, 96, 120, 144, 192, and 240 h post-administration.

Multiple-dose subcutaneous group (G6): Before the first dose (within 60 min prior to administration) and at 0.5, 2, 4, 8, 24, 48, 72, 96, 120, and 144 h post-administration; before the second to fourth administrations (within 2 min prior to administration) and at 24 h post-administration; and before the fifth administration (within 2 min prior to administration) and at 0.5, 2, 4, 8, 24, 48, 72, 96, 120, 144, 192, and 240 h post-administration.ADA blood sample collection time points were as follows:

G1-G5: At 0 h before administration (within 60 min prior to administration) and at 336, 504, and 672 h post-administration.

G6: Before the first dose (within 60 min prior to administration) and at 336, 672, 840, and 1,008 h post-administration.

### 2.2 Sample collection, processing, and storage

Blood samples of at least 1 mL were collected from the antecubital veins into 1.5 mL collection tubes containing EDTA-K2. The samples were inverted several times to ensure thorough mixing with EDTA-K2, then immediately centrifuged at 2000 g for 10 min at 2–8 °C at the sampling site. The plasma was collected and divided into two portions, each transferred to 1.5 mL EP tubes, and stored at −80 °C.

### 2.3 HDM7006 plasma concentration measurement

The concentration of HDM7006 in plasma was determined using a fully validated LC–MS/MS method. Plasma samples were processed by protein precipitation. Briefly, 50 μL of plasma sample were transferred to a 1.5 mL plastic centrifuge tube, followed by the addition of 200 μL of an internal standard working solution at 100 ng/mL. The mixture was vortexed at 2500 rpm for 3 min, then centrifuged at 4 °C for 4 min at 15,000 rpm. Then, 100 μL of the supernatant were mixed with 50 μL of 0.1% formic acid in methanol, vortexed again, and subjected to LC–MS/MS analysis.

The SCIEX QTRAP 6500 triple quadrupole LC–MS/MS instrument was used for sample analysis. The chromatographic column was the ACQUITY UPLC^®^ Peptide BEH C18 300 Å (2.1 × 100 mm, 1.7 μm), with a mobile phase consisting of water containing 0.2% formic acid (phase A) and a mixture of isopropanol and acetonitrile (50: 50, v: v) containing 5 mM of ammonium acetate (phase B). The flow rate was set at 0.300 mL/min, with the column temperature maintained at 50 °C; the autosampler temperature was 4 °C, and the injection volume was 10 μL. The linear LC gradient was as follows: time 0 min, 20% B; time 0.7 min, 20% B; time 1.0 min, 95% B; time 2.9 min, 95% B; time 2.91 min, 20% B; time 4.0 min, 20% B. Multiple reaction monitoring was used for MS analysis. The collision energy was set a 53 eV and 46 eV for quantitation with the following transitions: HDM7006: 1300.9 m/z → 1501.7 m/z, internal standard: 1264.6 m/z → 1453.5 m/z. Data were acquired in positive electrospray ionization (ESI) mode. The ion source had a gas temperature of 550 °C, collision gas pressure of medium, curtain gas pressure of 40 psi, nebulizer (GS1) and heater (GS2) gas pressure of 50 psi, and an ion spray voltage of 5,500 V. The linear range of the calibration curve was 10 to 10,000 ng/mL. The low limit of quantitation (LLOQ) was 10 ng/mL, and the signal-to-noise was more than 30. AB Sciex analysis software was used f or data analysis [SCIEX Analyst (V 1.7)]. The results were finally reported in units of ng/mL.

### 2.4 Measurement and analysis of ADA samples

A Bridging-ELISA method was established for the qualitative detection of anti-HDM7006 antibodies in cat plasma. The principle is as follows: a blank microplate was pre-coated with the test drug HDM7006. The anti-HDM7006 ADA in the test sample bound to it, and then Biotin-labeled HDM7006 was added, forming an “HDM7006 ~ ADA ~ Biotin-HDM7006” complex. Subsequently, HRP-labeled streptavidin (SA-HRP) was added. After washing the plate, TMB was added for color development, followed by a stop solution. The response was read at 450 nm using a microplate reader. The OD value was positively correlated with the anti-HDM7006 ADA concentration. The positive control samples were prepared with 100% normal healthy cat plasma.

Through comprehensive method validation, the screening cut point (SCP), titer cut point (TCP), confirmatory cut point (CCP), negative control acceptance criteria (NRC), and antibody response criteria (ARC) of the method were all acceptable. Its precision, sensitivity and hook effect, drug tolerance, titer, selectivity, stability, and robustness met the requirements for immunogenicity studies of clinical biologics.

The validated bridging ELISA method was used for the qualitative detection of anti-HDM7006 antibodies in cat plasma. A tiered testing strategy was employed, including screening, confirmatory, and titer assay. Each analytical batch included test samples and a series of system suitability samples such as negative control (NC), low positive control (LPC), high positive control (HPC), immunodepleted low positive control (LDTC), and immunodepleted high positive control (HDTC). The screening and confirmatory assay sensitivities were 88.0 ng/mL and 88.2 ng/mL, respectively. 250 ng/mL, 500 ng/mL and 1,000 ng/mL HDM7006 anti-drug antibody can tolerate 0 μg/mL, 1.6 μg//mL and 6.3 μg//mL HDM7006 in cat plasma.

### 2.5 Materials

The investigational drug HDM7006 injection was manufactured by Chengdu Shengnuo Biopharm Co., Ltd., upon commission from Hangzhou Zhongmei Huadong Pharmaceutical Co., Ltd., with a specification of 1 mL: 4 mg and an injection formulation.

The internal standard was ZMHD-P014, an analog of HDM7006, and manufactured by Chengdu Shengnuo Biopharm Co., Ltd., upon commission from Hangzhou Zhongmei Huadong Pharmaceutical Co., Ltd.

The HDM7006 anti-drug antibody is a rabbit polyclonal antibody obtained by subcutaneous injection of the HDM7006-KLH (keyhole limpet haemocyanin) conjugate into New Zealand white rabbits, and was purified using SDS (Sodium Dodecyl Sulfate)-PAGE (PolyAcrylamide Gel Electrophoresis). The anti-HDM7006 antibody was produced by Nanjing GenScript Biotechnology Co., Ltd., upon commission from Hangzhou Zhongmei Huadong Pharmaceutical Co., Ltd., with a concentration of 1.381 mg/mL.

The ADA capture reagent Bio-HDM7006 was labeled by United-Power Pharma Tech Co., Ltd. (UPP). The detection reagent peroxidase-Steptavidin (SA-HRP) was purchased by UPP from Jackson ImmunoResearch.

### 2.6 Animal selection

The study enrolled a total of 48 healthy British Shorthair cats of ordinary grade, aged 12–15 months and weighing 2.8–5.3 kg, with an equal gender distribution. The cats were randomly assigned to 6 groups (G1-G6), with 8 cats per group. One group received a single intravenous injection (G1); four groups received a single subcutaneous dose at different dose levels (G2-G5); and one group received a repeated-dose regimen (G6).

All recruited cats underwent disease screening and health status assessment and met the enrollment criteria before being admitted to the experimental facility. After completing quarantine and isolation procedures in the laboratory, the cats were assigned to experimental groups for study. All subjects were housed in individual cages and received standard feeding and water supply practices, adhering to established animal welfare protocols. In the event of stress responses or other adverse reactions during the experiment, veterinarians or laboratory personnel promptly provided care, nursing, and treatment in accordance with facility standard operating procedures (SOPs).

### 2.7 Statistical analysis

#### 2.7.1 PK parameters calculation

The pharmacokinetic parameters of the test drug were calculated using non-compartmental analysis (NCA) with WinNonlin (Version 8.3).

The main single-dose pharmacokinetic parameters included AUC_0→t_, AUC_0→∞_, C_max_, F, and T_max_. Dose-exposure proportionality was assessed for C_max_ and AUC after subcutaneous injection at different doses using the Power Model (48). One-way analysis of variance (ANOVA) was used for significance testing of the pharmacokinetic parameters C_max_ and AUC to study the relationship between C_max_ and AUC in animals of different genders. The absolute bioavailability (F) was calculated using the following formula.

$$F=\frac{{\mathrm{AUC}}_{0\to t\;\mathrm{sc}}\times {\text{Dose}}_{\mathrm{iv}}}{{\mathrm{AUC}}_{0\to t\;\mathrm{iv}}\times {\text{Dose}}_{\mathrm{sc}}}\times 100\%$$

AUC_0→t_ represents the area under the concentration-time curve, Dose represents the administered dose, and sc and iv represent subcutaneous and intravenous administration, respectively.

In multiple-dose pharmacokinetics, the main parameters after the first dose included T_max_, C_max_, AUC, T_1/2_, Vd, MRT, and CL. The parameters after the last dose included T_max_, C_min,ss_, C_max,ss_, C_av,ss_, T_1/2_, CL_ss_/F, V_ss_/F, AUC_ss_, degree of fluctuation (DF), and accumulation index (R_ac_).

The DF was calculated using the following formula.

$$\mathrm{DF}=\frac{C_{\max,\;\mathrm{ss}}-C_{\min,\mathrm{ss}}}{C_{\mathrm{av},\mathrm{ss}}}$$

The Rac was calculated using the following formula.

$$R_{\mathrm{ac}}=\frac{1}{|1-e^{-\mathrm{\lambda z}\times \tau }|}$$

#### 2.7.2 Statistical analysis

Watson LIMS™ 7.5SP1 or MS Office Excel was used for ADA sample and raw data management and analysis.

## 3 Results

### 3.1 Animal clinical observation

Single dose: a total of 40 cats were enrolled in the study, and 39 completed the study. In the 1.0 mg/kg single-dose group (G4), one female cat exhibited a significant stress reaction on Day 8 post-dose. Upon veterinary evaluation, the animal was deemed unsuitable for continued participation in the study and was excluded per protocol. The remaining 39 cats exhibited target-related anorexia as their main clinical manifestation, with no other adverse reactions observed.

Multiple doses: a total of 8 cats were enrolled in the study, with 7 completing the study. One female cat exhibited significant stress reaction after receiving 0.5 mg/kg (G6) of HDM7006 for 5 days. Following veterinary evaluation, the animal was excluded per protocol. The remaining 7 cats primarily exhibited target-related anorexia throughout the study, with no other adverse reactions observed.

### 3.2 Single dose PK

The concentration-time curve for a single intravenous injection is shown in Figure 1. PK parameter statistics are presented in Tables 1–4, including exposure comparisons between males and females (Table 3) and dose-exposure relationships (Table 4).

**Figure 1:**
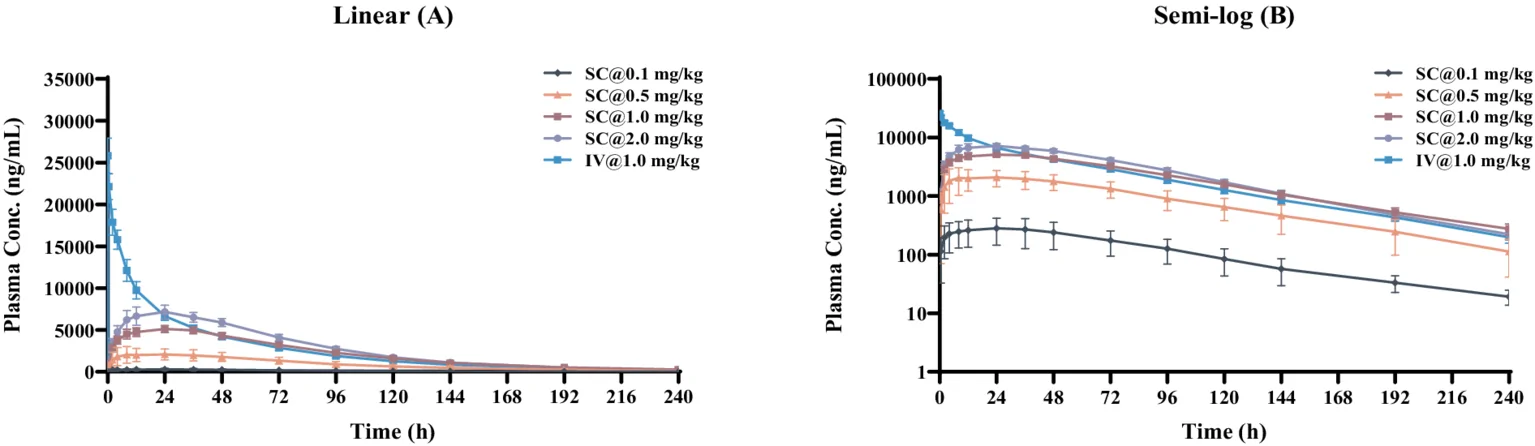
Mean Concentration-Time Curve of HDM7006 after a single dose. The left is a linear plot and the right a semi-logarithmic plot.

**Table 1:** Pharmacokinetic parameters of HDM7006 injection after intravenous injection.

Parameter	Unit	Sex	Intravenous Injection 1.0 mg/kg (*n* = 8, 4 females/4 males)
λz	l/h	Female	0.0170 ± 0.00261 (15.35%)
		Male	0.0155 ± 0.00295 (19.03%)
		Female + Male	0.0162 ± 0.00269 (16.58%)
T_1/2_	h	Female	41.6 ± 6.37 (15.31%)
		Male	45.8 ± 7.86 (17.16%)
		Female + Male	43.7 ± 7.00 (16.02%)
T_max*_	h	Female	0.0833 (0.0833,0.0833)
		Male	0.0833 (0.0833,0.0833)
		Female + Male	0.0833 (0.0833,0.0833)
C_max_	ng/mL	Female	22,750 ± 4,638 (20.39%)
		Male	28,875 ± 6,115 (21.18%)
		Female + Male	25,812 ± 5,997 (23.23%)
C_0_	ng/mL	Female	23,306 ± 4,778 (20.50%)
		Male	29,958 ± 6,593 (22.01%)
		Female + Male	26,632 ± 6,407 (24.06%)
AUC_0→t_	h·ng/mL	Female	581,334 ± 201,819 (34.72%)
		Male	725,664 ± 141,817 (19.54%)
		Female + Male	653,499 ± 178,962 (27.39%)
AUC_0→∞_	h·ng/mL	Female	590,454 ± 208,315 (35.28%)
		Male	743,392 ± 150,047 (20.18%)
		Female + Male	666,923 ± 186,895 (28.02%)
Vd	mL/kg	Female	109 ± 27.1 (24.86%)
		Male	89.8 ± 10.8 (12.03%)
		Female + Male	99.2 ± 21.6 (21.77%)
CL	mL/h/kg	Female	1.85 ± 0.576 (31.14%)
		Male	1.39 ± 0.278 (20.00%)
		Female + Male	1.62 ± 0.486 (30.00%)
MRT_0→t_	h	Female	46.5 ± 4.47 (9.61%)
		Male	50.0 ± 2.97 (5.94%)
		Female + Male	48.2 ± 3.98 (8.26%)
MRT_0→∞_	h	Female	50.0 ± 6.38 (12.76%)
		Male	55.9 ± 5.81 (10.39%)
		Female + Male	53.0 ± 6.45 (12.17%)

^*^T_max_ is expressed as median (minimum, maximum), other parameters are arithmetic mean ± SD (CV%).

**Table 2:** PK parameters of HDM7006 injection after single subcutaneous doses.

Parameter	Unit	Sex	Subcutaneous injection			
			0.1 mg/kg bw (*n* = 8, 4 females/4 males)	0.5 mg/kg bw (*n* = 8, 4 females/4 males)	1.0 mg/kg bw (*n* = 7, 3 females/4 males)	2.0 mg/kg bw (*n* = 8, 4 females/4 males)
λ_z_	1/h	Female	0.0174 ± 0.00341 (19.60%)	0.0151 ± 0.00180 (11.92%)	0.0169 ± 0.00348 (20.59%)	0.0191 ± 0.00382 (20.00%)
		Male	0.0158 ± 0.00314 (19.87%)	0.0147 ± 0.00228 (15.51%)	0.0146 ± 0.00414 (28.36%)	0.0165 ± 0.00153 (9.27%)
		Female + Male	0.0166 ± 0.00314 (18.92%)	0.0149 ± 0.00191 (12.82%)	0.0156 ± 0.00374 (23.97%)	0.0178 ± 0.00302 (16.97%)
T_1/2_	h	Female	41.1 ± 8.03 (19.54%)	46.4 ± 5.35 (11.53%)	42.2 ± 8.40 (19.91%)	37.4 ± 7.73 (20.67%)
		Male	45.3 ± 10.3 (22.74%)	47.8 ± 7.72 (16.15%)	49.9 ± 11.9 (23.85%)	42.2 ± 3.98 (9.43%)
		Female + Male	43.2 ± 8.83 (20.44%)	47.1 ± 6.20 (13.16%)	46.6 ± 10.5 (22.53%)	39.8 ± 6.23 (15.65%)
T_max*_	h	Female	24.0 (24.0, 36.0)	18.0 (4.00, 24.0)	24.0 (12.0, 36.0)	24.0 (12.0, 48.0)
		Male	14.0 (2.00, 36.0)	28.0 (4.00, 72.0)	24.0 (8.00, 48.0)	18.0 (12.0,48.0)
		Female + Male	24.0 (2.00, 36.0)	18.0 (4.00, 72.0)	24.0 (8.00, 48.0)	24.0 (12.0, 48.0)
C_max_	ng/mL	Female	281 ± 193 (68.68%)	2,155 ± 769 (35.68%)	5,893 ± 462 (7.84%)	7,622 ± 3,751 (49.21%)
		Male	314 ± 98.1 (31.24%)	2,708 ± 1,169 (43.17%)	5,062 ± 1,413 (27.91%)	7,480 ± 1,359 (18.17%)
		Female + Male	297 ± 143 (48.15%)	2,431 ± 962 (39.57%)	5,419 ± 1,125 (20.76%)	7,551 ± 2,613 (34.60%)
AUC_0→t_	h·ng/mL	Female	25,098 ± 17,927 (71.43%)	187,044 ± 79,681 (42.60%)	511,152 ± 120,139 (23.50%)	584,955 ± 234,890 (40.16%)
		Male	30,090 ± 8,223 (27.33%)	240,060 ± 40,384 (16.82%)	513,039 ± 113,844 (22.19%)	702,466 ± 122,620 (17.46%)
		Female + Male	27,594 ± 13,185 (47.78%)	213,552 ± 64,985 (30.43%)	512,230 ± 106,265 (20.75%)	643,710 ± 184,485 (28.66%)
F	%	Female	43.17	64.35	87.93	50.31
		Male	41.47	66.16	70.70	48.40
		Female + Male	42.23	65.37	78.38	49.25

^*^T_max_ is expressed as median (minimum, maximum), other parameters are arithmetic mean ± SD (CV%).

**Table 3:** Ratio of mean pharmacokinetic parameters between female and male animals in each dose group.

Group	Parameter	Unit	Female	Male	Female/Male	*p*-value
G1-IV@1.0 mg/kg	C_0_	ng/mL	23,306	29,958	0.778	0.153
	AUC_0→t_	h·ng/mL	581,334	725,664	0.801	0.286
	AUC_0→∞_	h·ng/mL	590,454	743,392	0.794	0.278
G2-SC@0.1 mg/kg	C_max_	ng/mL	281	314	0.895	0.776
	AUC_0→t_	h·ng/mL	25,098	30,090	0.834	0.631
	AUC_0→∞_	h·ng/mL	26,378	31,297	0.843	0.638
G3- SC@0.5 mg/kg	C_max_	ng/mL	2,155	2,708	0.796	0.460
	AUC_0→t_	h·ng/mL	187,044	240,060	0.779	0.280
	AUC_0→∞_	h·ng/mL	193,106	250,332	0.771	0.274
G4- SC@1.0 mg/kg	C_max_	ng/mL	5,893	5,062	1.16	0.381
	AUC_0→t_	h·ng/mL	511,152	513,039	0.996	0.984
	AUC_0→∞_	h·ng/mL	526,053	537,626	0.978	0.907
G5- SC@2.0 mg/kg	C_max_	ng/mL	7,622	7,480	1.02	0.945
	AUC_0→t_	h·ng/mL	584,955	702,466	0.833	0.409
	AUC_0→∞_	h·ng/mL	594,508	720,159	0.826	0.395

**Table 4:** Analysis of the linear relationship between pharmacokinetic parameters and dose.

Parameter	Dose range	Slope	90% confidence interval	Determination interval
C_max_	0.1–2.0 mg/kg bw	1.12	1.04 ~ 1.20	0.77 ~ 1.23
AUC_0→t_	0.1–2.0 mg/kg bw	1.10	1.02 ~ 1.18	0.77 ~ 1.23

One-way ANOVA was performed on the main pharmacokinetic parameters C_0_ or C_max_, AUC, etc., for male and female animals within the single intravenous and single subcutaneous injection groups. The results showed no statistically significant differences in the main pharmacokinetic parameters between male and female animals in each dose group (*p* > 0.05) (Table 3), and the parameter ratios between male and female animals in each dose group were all within the range of 0.5–2.0. This indicates that there were no gender differences in HDM7006 exposure in animals in the different single-dose groups, so the data from male and female animals were combined for analysis.

After a single intravenous injection of 1.0 mg/kg, the initial concentration C_0_ after intravenous injection was 26,632 ± 6,407 ng/mL, the elimination half-life T_1/2_ was 43.7 ± 7.00 h, and AUC_0→t_ and AUC_0→∞_ were 653,499 ± 178,962 h·ng/mL and 666,923 ± 186,895 h·ng/mL, respectively. The CL was 1.62 mL/h/kg, significantly lower than 30% of the hepatic blood flow in cats (1,560 mL/h/kg) (49). The V_d_ was 99.2 mL/kg, slightly greater than the total blood volume in cats (~70 mL/kg) (Table 1).

After single SC doses (0.1, 0.5, 1.0, and 2.0 mg/kg bw), the median T_max_ of HDM7006 was 18.0–24.0 h, and the elimination half-life T_1/2_ was 39.8–47.1 h. The absolute bioavailability (F) at doses of 0.1, 0.5, 1.0, and 2.0 mg/kg was 42.23%, 65.37%, 78.38%, and 49.25%, respectively (Table 2). A dose-exposure proportionality analysis was performed on C_max_ and AUC with the Power Model. Linear regression was performed after natural logarithmic transformation of C_max_, AUC, and dose to calculate the slope and 90% confidence interval. For both C_max_ and AUC, the determination interval was calculated using *θ_L_* = 0.5 and *θ_H_* = 2.0. The results are presented in Table 4 and showed that the 90% confidence interval of the slope fell within the determination interval of 0.77–1.23 in the dose range of 0.1–2.0 mg/kg. Therefore, HDM7006 exhibits linear pharmacokinetic characteristics in the dose range of 0.1–2.0 mg/kg, indicating proportional increases in exposure with dose, with no significant saturation or inhibition.

### 3.3 Multiple-dose PK

PK parameters after the first and final administration of 0.5 mg/kg HDM7006 injection administered once weekly to cats (total five doses) are presented in Tables 5, 6. The concentration-time curves are shown in Figure 2. The comparisons of exposure levels between males and females are shown in Table 7, and trough concentrations before and 24 h post-administration are shown in Table 8.

**Table 5:** Pharmacokinetic parameters after the first dose of HDM7006 injection.

Parameter	Unit	Sex	First Dose at 0.5 mg/kg bw (*n* = 7, 3 females/4 males)
λz	1/h	Female	0.0143 ± 0.00155 (10.84%)
		Male	0.0122 ± 0.00208 (17.05%)
		Female + Male	0.0131 ± 0.00207 (15.80%)
T_1/2_	h	Female	48.6 ± 5.01 (10.31%)
		Male	58.0 ± 8.95 (15.43%)
		Female + Male	54.0 ± 8.61 (15.94%)
T_max*_	h	Female	24.0 (2.00,24.0)
		Male	48.0 (48.0,48.0)
		Female + Male	48.0 (2.00,48.0)
C_max_	ng/mL	Female	2,447 ± 1,235 (50.47%)
		Male	1868 ± 335 (17.93%)
		Female + Male	2,116 ± 813 (38.42%)
AUC_0→t_	h·ng/mL	Female	204,825 ± 100,260 (48.95%)
		Male	213,717 ± 42,041 (19.67%)
		Female + Male	209,906 ± 65,246 (31.08%)
AUC_0→∞_	h·ng/mL	Female	227,397 ± 105,767 (46.51%)
		Male	262,584 ± 57,334 (21.83%)
		Female + Male	247,504 ± 75,672 (30.57%)
V_d_/F	mL/kg	Female	187 ± 108 (57.75%)
		Male	162 ± 25.8 (15.93%)
		Female + Male	173 ± 66.5 (38.44%)
CL/F	mL/h/kg	Female	2.59 ± 1.31 (50.58%)
		Male	1.97 ± 0.375 (19.04%)
		Female + Male	2.23 ± 0.868 (38.92%)
MRT_0→t_	h	Female	60.9 ± 1.46 (2.40%)
		Male	74.0 ± 2.41 (3.26%)
		Female + Male	68.4 ± 7.29 (10.66%)
MRT_0→∞_	h	Female	79.9 ± 6.31 (7.90%)
		Male	107 ± 8.32 (7.78%)
		Female + Male	95.4 ± 16.1 (16.88%)

*T_max_ is expressed as median (minimum, maximum), other parameters are arithmetic mean ± SD (CV%).

**Table 6:** Pharmacokinetic parameters after the last dose of HDM7006 injection.

Parameter	Unit	Sex	Last Dose at 0.5 mg/kg bw (*n* = 7, 3 females/4 males)
λz	1/h	Female	0.0152 ± 0.00220 (14.47%)
		Male	0.0124 ± 0.00142 (11.45%)
		Female + Male	0.0136 ± 0.00219 (16.10%)
T_1/2_	h	Female	46.4 ± 6.71 (14.46%)
		Male	56.3 ± 6.30 (11.19%)
		Female + Male	52.1 ± 7.94 (15.24%)
T_max*_	h	Female	24.0 (4.00,24.0)
		Male	24.0 (24.0,72.0)
		Female + Male	24.0 (4.00,72.0)
C_max,ss_	ng/mL	Female	2,163 ± 1,061 (49.05%)
		Male	2,225 ± 283 (12.72%)
		Female + Male	2,199 ± 645 (29.33%)
C_min,ss_	ng/mL	Female	384 ± 156 (40.62%)
		Male	739 ± 155 (20.97%)
		Female + Male	587 ± 237 (40.37%)
C_av,ss_	ng/mL	Female	1,150 ± 564 (49.04%)
		Male	1,555 ± 180 (11.58%)
		Female + Male	1,381 ± 411 (29.76%)
AUC_0→t,ss_	h·ng/mL	Female	207,849 ± 98,779 (47.52%)
		Male	295,269 ± 37,654 (12.75%)
		Female + Male	257,803 ± 78,389 (30.41%)
AUC_0→∞,ss_	h·ng/mL	Female	215,078 ± 99,158 (46.10%)
		Male	318,661 ± 45,794 (14.37%)
		Female + Male	274,268 ± 85,974 (31.35%)
AUC_0→tau.ss_	h·ng/mL	Female	193,160 ± 94,722 (49.04%)
		Male	261,266 ± 30,150 (11.54%)
		Female + Male	232,078 ± 69,069 (29.76%)
V_ss_/F	mL/kg	Female	204 ± 95.0 (46.57%)
		Male	156 ± 10.7 (6.86%)
		Female + Male	176 ± 61.1 (34.72%)
CL_ss_/F	mL/h/kg	Female	2.96 ± 1.14 (38.51%)
		Male	1.93 ± 0.217 (11.24%)
		Female + Male	2.37 ± 0.868 (36.62%)
MRT_0→∞_	h	Female	83.6 ± 8.41 (10.06%)
		Male	107 ± 11.6 (10.84%)
		Female + Male	97.0 ± 15.8 (16.29%)
DF	%	Female	154.85 ± 36.06 (23.29%)
		Male	96.18 ± 14.65 (15.23%)
		Female + Male	121.32 ± 39.04 (32.18%)
R_ac_	/	Female	1.09 ± 0.0351 (3.22%)
		Male	1.15 ± 0.0403 (3.50%)
		Female + Male	1.12 ± 0.0454 (4.05%)

^*^T_max_ is expressed as median (minimum, maximum), other parameters are arithmetic mean ± SD (CV%).

**Figure 2:**
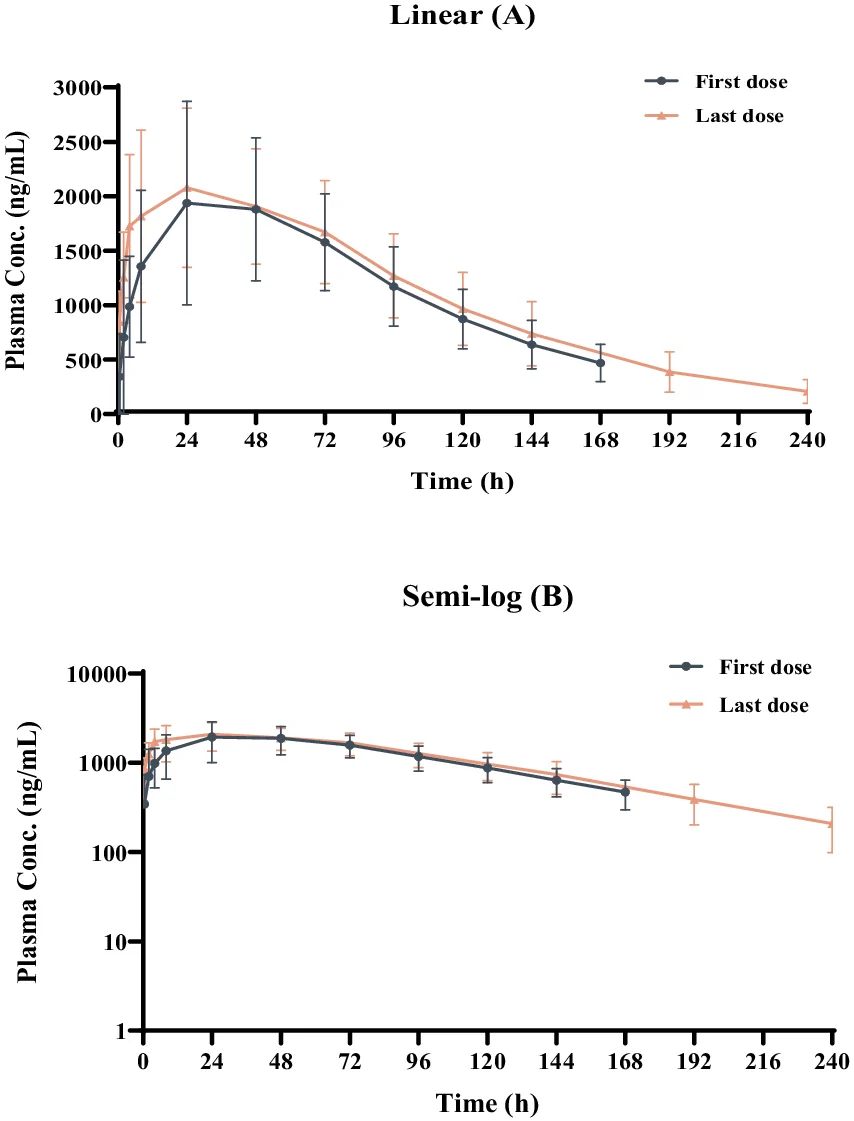
Mean Concentration-Time Curve of HDM1005 after the First and Last Dose. The top is a linear plot and the bottom a semi-logarithmic plot.

**Table 7:** Ratio of mean pharmacokinetic parameters between female and male animals after the first and last doses.

Dose	Parameter	Unit	Female	Male	Female/Male	*p*-value
First	C_max_	ng/mL	2,447	1868	1.31	0.399
	AUC_0→t_	h·ng/mL	204,825	213,717	0.958	0.877
	AUC_0→∞_	h·ng/mL	227,397	262,584	0.866	0.591
Last	C_max,ss_	ng/mL	2,163	2,225	0.972	0.913
	AUC_0→t,ss_	h·ng/mL	207,849	295,269	0.704	0.158
	AUC_0→∞,ss_	h·ng/mL	215,078	318,661	0.675	0.119

**Table 8:** Pre-dose trough plasma concentration and 24 h post-dose concentration data.

Number of doses	Pre-dose trough plasma concentration C_trough_ (ng/mL)	24 h post-dose concentration (ng/mL)
Dose 1	0	1937 ± 934
Dose 2	468 ± 172	2,219 ± 852
Dose 3	547 ± 208	2,333 ± 899
Dose 4	608 ± 212	2,357 ± 720
Dose 5	597 ± 226	2080 ± 731

Consistent with the single-dose PK results, no gender differences in exposure levels were observed in repeated dosing studies (Table 7). Therefore, the combined gender parameters were used for further analysis.

After multiple subcutaneous injections of 0.5 mg/kg HDM7006 Injection in cats, the time to peak concentration T_max_ after the first and last doses were 48.0 (2.00, 48.0) h and 24.0 (4.00, 72.0) h, respectively. The elimination half-life T_1/2_ was 54.0 ± 8.61 h and 52.1 ± 7.94 h, respectively. The plasma peak concentration C_max_ after the first dose and the plasma peak concentration at steady state C_max,ss_ after the last dose were 2,116 ± 813 ng/mL and 2,199 ± 645 ng/mL, respectively. The clearance after the first dose (CL/F) and the clearance at steady state after the last dose (CL_ss_/F) were 2.23 ± 0.868 mL/h/kg and 2.37 ± 0.868 mL/h/kg, respectively. After multiple doses, AUC_0→t_ after the first dose and AUC_0→tau,ss_ after the last dose were 209,906 ± 65,246 h·ng/mL and 232,078 ± 69,069 h·ng/mL, respectively. The three consecutive trough plasma concentrations C_trough_ measured before the last dose ranged from 547 to 608 ng/mL. The accumulation index R_ac_ after multiple doses was 1.12 ± 0.0454, which indicates no significant accumulation of HDM7006 after multiple doses (50).

### 3.4 Immunogenicity

The analysis results of the ADA samples are present in Table 9. Following a tiered testing including screening, confirmatory, and titer assays, 2 out of 152 samples from the single-dose study were confirmed positive, yielding an ADA positive rate of 1.33%; notably, both positive results came from the 336 h post-dose samples of a single test cat. In contrast, all 35 samples collected from multiple-dose ADA study were negative. These results indicate a low immunogenic potential of HDM7006 in cats.

**Table 9:** Summary of ADA test results.

Assays	Results
Screening assay	No. of samples tested: 193	
	No. of negative screening samples: 181	
	No. of positive screening samples: 12	
Confirmatory assay	No. of samples tested: 12	
	No. of negative confirmatory samples: 10	
	No. of positive confirmatory samples: 2	
Titer assay	No. of samples tested: 2	
Sample re-analysis	Total number of samples re-analyzed: 23	
Results included in statistical analysis	Single dose (152)	Negative samples: 150; positive samples: 2 Positive rate: 1.33%
	Multiple doses (35)	Negative samples: 35; positive samples: 0 Positive rate: 0%

## 4 Discussion and conclusion

GLP-1R/GIPR dual agonists have been approved for T2D and long-term weight management in obese/overweight adult, in seven countries including the United States, the European Union, and China, demonstrating favorable clinical efficacy and market value in both indications (51). The HDM7006, a GLP-1R/GIPR dual agonist developed for veterinary usage, has shown superior glycemic control and weight management efficacy with favorable safety profiles to Tirzepatide in preclinical studies (52). HDM7006 has entered Phase III clinical trials for both adult T2D treatment and long-term weight management in obese/overweight adults (45–47). To date, no weight-loss drug has been approved for use in pet cats, although several studies have reported the application of GLP-1 analogues applied in cats. Exenatide (53–55) was reported to induce significant weight loss in healthy cats under chronic administration at 1 μg/kg twice daily. Another GLP-1 analog, liraglutide (56), was reported had a similar effects and pharmacokinetics profile in cats to as in human, with a half-life of approximately 12 h. However, to date, exenatide and liraglutide were not approved for type 2 diabetes in cats. The detailed reasons have not been publicly reported. We noticed that one study of Clinical Evaluation of a Novel GLP1 Agonist for Weight Loss in Overweight and Obese Client-Owned Domestic Cats was sponsored by Akston Biosciences at College of Cornell University. The studied period was July 2025 to June 2026 (57). The first domestically developed GLP-1 receptor agonist approved in China, Efsubaglutide α, has received formal acceptance from the Ministry of Agriculture and Rural Affairs of the People’s Republic of China for its new veterinary drug clinical trial application to treat pet diabetes. Phase I clinical trials commenced in the first quarter of 2026 (58). Furthermore, in a concurrent clinical study, we have demonstrated the excellent weight reduction efficacy and safety profile of HDM7006 in obese cats (Author Zhiping Zhang, Li Teng, Shujun Cui, *et al*., submitted for publication). Together with the favorable pharmacokinetic profile observed in the present study, these findings establish HDM7006 as a highly promising long-term weight management agent in felines.

To support the clinical study of HDM7006, including the subcutaneous titration regimen employed in Phase III trials (0.1 to 0.5 mg/kg), this study comprehensively evaluated its PK profile across a broad dose range (0.1–2.0 mg/kg). An intravenous group was also included to determine the absolute bioavailability of subcutaneous administration. Following a single intravenous dose in cats, HDM7006 exhibited typical PK characteristics of peptide drugs, including low clearance rate (CL: 1.62 mL/h/kg) and a long half-life (T_1/2_: 43.7 h). After a single subcutaneous dose within the 0.1–2.0 mg/kg range, no gender differences were observed in exposure levels, with essentially linear PK profiles. The half-life supported a weekly dosing frequency (T_1/2_: 39.8–47.1 h), and the drug demonstrated favorable bioavailability (%F: 42.23–78.38%). This study also included multiple subcutaneous PK studies in healthy cats using a clinical targeted maintenance dose of 0.5 mg/kg administered once weekly for five doses, exceeding the targeted maintenance dose frequency by three doses. According to classical pharmacokinetic theory, steady-state concentrations are achieved after 4–6 half-lives. In this study, steady-state trough concentrations were essentially reached after approximately three doses. No significant difference was observed in exposure levels between the first, second, and final administration, indicating that HDM7006 does not accumulate in cats following repeated dosing.

In December 2023, the U. S. FDA issued a key guideline for peptide drug products titled Clinical Pharmacology Considerations for Peptide Drug Products (59). This guideline emphasizes the importance of immunogenicity risk assessment for peptide drugs, urging researchers to enhance their attention to potential immunogenic risks associated with peptide-based therapeutics. HDM7006, a peptide drug comprising 46 amino acids, was evaluated for its immunogenicity in this study. The results demonstrated that HDM7006 exhibited a low positive rate for ADA production in cats, with only 2 out of 152 samples testing positive (1.33%) following single-dose administration, and both positive samples exhibited low titers (titers of 20) in a single cat. More importantly, no ADA was detected following repeated weekly dosing. Therefore, HDM7006 poses a low immunogenicity risk in cats.

In both single-dose (1.0 mg/kg) and multiple-dose (0.5 mg/kg subcutaneous, once-weekly dosing for a total of 5 doses) PK studies, one cat withdrew due to stress responses. This may be related to the inherent characteristics of cats. Cats exhibit greater emotional complexity compared to typical laboratory animals and are more sensitive to certain experimental procedures, often manifesting symptoms such as urinary retention, anorexia, and depression. In this study, HDM7006 itself possesses appetite-suppressing pharmacological effects, and the administration and blood sampling procedures may have exacerbated stress responses in the cats.

In summary, this study comprehensively evaluated the PK characteristics of HDM7006 in cats. HDM7006 exhibits favorable PK profiles, with an adequate half-life supporting once-weekly subcutaneous administration, warranting further investigation of HDM7006 in diabetic and overweight cats.

## Data Availability

The original contributions presented in the study are included in the article/supplementary material, further inquiries can be directed to the corresponding author.
